# Costs and assessment in medical education: a strategic view

**DOI:** 10.1007/s40037-016-0299-8

**Published:** 2016-09-14

**Authors:** Kieran Walsh

**Affiliations:** BMJ Learning, BMJ Publishing Group, London, UK

Van der Vleuten and Heeneman have added another important piece to the emerging jigsaw that will eventually show us the full picture of cost in healthcare professional education [1]. Their idea of redistributing the resources of assessment in a curriculum is an attractive one. Their suggestion that we concentrate more on progress testing could make a real difference in how we allocate funding to different forms of assessment. Perhaps more importantly it could also make a real difference to the outcomes that we can achieve by means of assessment for a given cost. Inevitably there are gaps in their approach. Their estimates of costs are in their own words ‘rough’ and there is also limited data. However, the main strength of their approach is that they bring much needed radical thinking to the area of cost in assessment. In this short commentary I will try to articulate some more radical thinking in this field.

The issue of cost and value in healthcare professional education generally and assessment in healthcare professional education specifically has been largely neglected until relatively recently. There has been little research and few systematic reviews. Academic endeavours in this field have not always been of high quality. Terms like cost-effectiveness are used loosely and sometimes lazily: interventions are described as cost-effective without a thorough analysis of their costs or their effectiveness or a comparison with other interventions of differing cost-effectiveness [2; 3]. Put simply, no intervention can be cost-effective in and of itself – it can only be considered to be cost-effective in comparison to an alternative. In the absence of such comparisons conclusions are merely rhetoric.

A proper cost-effectiveness analysis ‘refers to the evaluation of two or more alternative approaches or interventions according to their costs and their effects in producing a certain outcome’ [4]. Cost is rarely referred to directly; we tend to prefer the pusillanimous word feasibility. Research studies on cost that have emerged are a mélange of different types, even though that is both a strength and a weakness [5; 6]. The strength is that it has allowed us to look at a range of interventions in a range of ways, which is important in an emerging field. However the weakness is that the resultant conclusions can sound like a cacophony and that sometimes there has been too close a focus on specific and small interventions that we might be able deliver in a more cost-effective way but, even if this were to happen, the cost savings would be minimal. Let’s look at the following tangible example. It is fictional but hopefully will find some resonance with readers.

Say a department decides to produce an e‑learning resource on a clinical subject for year 4 students. The team debate what format it should take and realize that different formats will have different costs. They decide to do a cost-effectiveness analysis to compare the relative cost-effectiveness of a simple video programme and a sophisticated interactive multimedia programme. At the end of the study they find that both programmes are equally popular and are equally used and that students who take them have similar test results at the end. The cost of the simple programme is £7000 and the cost of the multimedia programme is £10,000. They correctly conclude that the simple programme is more cost-effective.

But, looking at the issue critically, what have they really achieved? They have gone to considerable effort to achieve cost savings of £3000. In the context of healthcare professional education budgets this is a nugatory amount of money [7]. This saving might be scalable – they and others may be able to apply their approach to other fields – but equally they may not. What works in one clinical field may not work in another: learning and assessment are context specific [8]. In reality they have not thought radically, and there is still too much research like this in the healthcare professional education field more broadly but also in the specialized field of research into cost and value in healthcare professional education.

There are other approaches to looking at this problem. We could look critically at parts of the curriculum that are larger and more expensive and where a small percentage reduction in costs could result in significant savings in real terms. To adopt this approach we need to stand back and look at the wider landscape. So as Van der Vleuten and Heeneman suggest, we could look at objective structured clinical examinations (OSCEs). These are expensive and so should only be used where they add real value to assessment [9]. We could stand further back and look at assessment as a whole and its relation to the rest of the curriculum and how it could be better integrated into the curriculum. The end-result might be a saving of separate costs for assessment – as assessment and learning become part of a greater whole. Standing further back still we could look at the outcomes that we are trying to achieve through healthcare professional education programmes and the fiscally optimal methods of achieving these outcomes. This might mean investment in short postgraduate programmes that help doctors in training to credential in different areas. The result might be more flexible programmes and more flexible healthcare professionals. Healthcare professional education is long and complicated – we are trying to educate young people to be the fully qualified professionals that the country will need in five or ten years’ time and yet we don’t know what the future will hold. The only certainty is uncertainty. However, a more flexible workforce should be better able to cope with uncertainty and the development of such a workforce need not be more expensive than the way that we do things today. We should also think about who bears the costs of healthcare professional education. Is it the learner, the institution or the government? Different stakeholders are likely to value different outcomes in different ways. Thinking through the issues from a completely different perspective might lead us to consider healthcare professionals in training as a resource rather than a cost burden. Encouraging these healthcare professionals to learn about quality improvement at the same time as they learn how to measure and improve quality is one such example of how this could work in practice [10; 11]. The assessment of their quality improvement activities could be related to the actual progress that they have made in improving quality, with of course the caveat that not all quality improvement projects succeed despite being managed to high standards.

Commentaries in healthcare professional education often end with a call for more research. In this case I think that we need more profound and innovative thinking to develop new concepts and ideas in the field of cost and value in healthcare professional education. Then and only then should we test these ideas. The good news is that, even though our thinking is currently inchoate, healthcare professional education offers much fecund ground for further and more profound deliberation.
